# Intracranial-to-central venous pressure gap predicts the responsiveness of intracranial pressure to PEEP in patients with traumatic brain injury: a prospective cohort study

**DOI:** 10.1186/s12883-020-01764-7

**Published:** 2020-06-08

**Authors:** Hong Peng Li, Ying Ni Lin, Zhi Hui Cheng, Wei Qu, Liu Zhang, Qing Yun Li

**Affiliations:** 1 Department of Respiratory and Critical Care Medicine, Ruijin Hospital, Shanghai Jiao Tong University School of Medicine, Shanghai, 200025 People’s Republic of China; 2 Institute of Respiratory Medicine, Shanghai Jiao Tong University School of Medicine, Shanghai, 200025 People’s Republic of China; 3 Department of Emergency and Critical Care Medicine, Shanghai University of Medicine & Health Sciences Affiliated Zhoupu Hospital, Shanghai, 201318 People’s Republic of China

**Keywords:** Positive end-expiratory pressure, Intracranial pressure, Central venous pressure, Mechanical ventilation, Traumatic brain injury, P_IC_Gap

## Abstract

**Background:**

Mechanical ventilation (MV) with positive end-expiratory pressure (PEEP) is commonly applied in patients with severe traumatic brain injury (sTBI). However, the individual responsiveness of intracranial pressure (ICP) to PEEP varies. Thus, identifying an indicator detecting ICP responsiveness to PEEP is of great significance. As central venous pressure (CVP) could act as an intermediary to transduce pressure from PEEP to ICP, we developed a new indicator, P_IC_Gap, representing the gap between baseline ICP and baseline CVP. The aim of the current study was to explore the relationship between P_IC_Gap and ICP responsiveness to PEEP.

**Methods:**

A total of 112 patients with sTBI undergoing MV were enrolled in this prospective cohort study. ICP, CVP, cerebral perfusion pressure (CPP), static compliance of the respiratory system (Cst), and end-tidal carbon dioxide pressure (PetCO_2_) were recorded at the initial (3 cmH_2_O) and adjusted (15 cmH_2_O) levels of PEEP. P_IC_Gap was assessed as baseline ICP - baseline CVP (when PEEP = 3 cmH_2_O). The patients were classified into the ICP responder and non-responder groups based on whether ICP increment with PEEP adjusted from 3 cmH_2_O to 15 cmH_2_O was greater than 20% of baseline ICP. The above parameters were compared between the two groups, and prediction of ICP responsiveness to PEEP adjustment was evaluated by receiver operating characteristic (ROC) curve analysis.

**Results:**

Compared with the non-responder group, the responder group had lower P_IC_Gap (1.63 ± 1.33 versus 6.56 ± 2.46 mmHg; *p* <  0.001), lower baseline ICP, and higher baseline CVP. ROC curve analysis suggested that P_IC_Gap was a stronger predictive indicator of ICP responsiveness to PEEP (AUC = 0.957, 95%CI 0.918–0.996; *p* <  0.001) compared with baseline ICP and baseline CVP, with favorable sensitivity (95.24, 95%CI 86.91–98.70%) and specificity (87.6, 95%CI 75.76–94.27%), at a cut off value of 2.5 mmHg.

**Conclusion:**

The impact of PEEP on ICP depends on the gap between baseline ICP and baseline CVP, i.e. P_IC_Gap. In addition, P_IC_Gap is a potential predictor of ICP responsiveness to PEEP adjustment in patients with sTBI.

## Background

Mechanical ventilation (MV) with positive end-expiratory pressure (PEEP) is often required in patients with severe traumatic brain injury (sTBI) due to neurologic, airway, and pulmonary dysfunctions [1, 2]. Application of PEEP during MV is essential to improving oxygenation and protecting from mechanical lung injury, by increasing functional residual capacity, preventing atelectasis, reducing oxygen requirement, and raising static strain component [3–5]. .

However, the influence of PEEP on intracranial pressure (ICP) has been an obstacle to the optimal use of PEEP for a long time [6]. The effect of PEEP on ICP was first mentioned in the later 1970s [7, 8]. In the last three decades, several studies have explored the relationship between PEEP and ICP, but without consistent results [1]. Shapiro et al. demonstrated that PEEP application in the 4–8 cm H_2_O range increases ICP (> 10 mmHg) [7]. Flexman and colleagues also found that alveolar recruitment maneuvers increase subdural pressure and reduce cerebral perfusion pressure (CPP) in neurosurgery [9]. A recent study by Boone et al. found that every centimeter H_2_O increase of PEEP contributes to a 0.31 mmHg increase in ICP [10], and concluded that PEEP might exert adverse effects on cerebral hemodynamics by impeding cerebral venous return and elevating ICP in patients with sTBI. However, other studies found no effects of moderate to high levels of PEEP (8–25 cmH_2_O) on ICP, CPP, and cerebral blood flow (CBF) in sTBI patients with normal ICP or intracranial hypertension, and PEEP instead exerted favorable effects by improving brain tissue oxygen pressure and saturation [11–14]. Such discrepancy might be related to several factors: (1) individual heterogeneity, mainly involving the severity of and baseline ICP [15]; (2) not fully understood dose-effect relationship between PEEP and ICP; and (3) it is unclear whether PEEP directly affects ICP or indirectly through an intermediate.

**Fig. 1:**
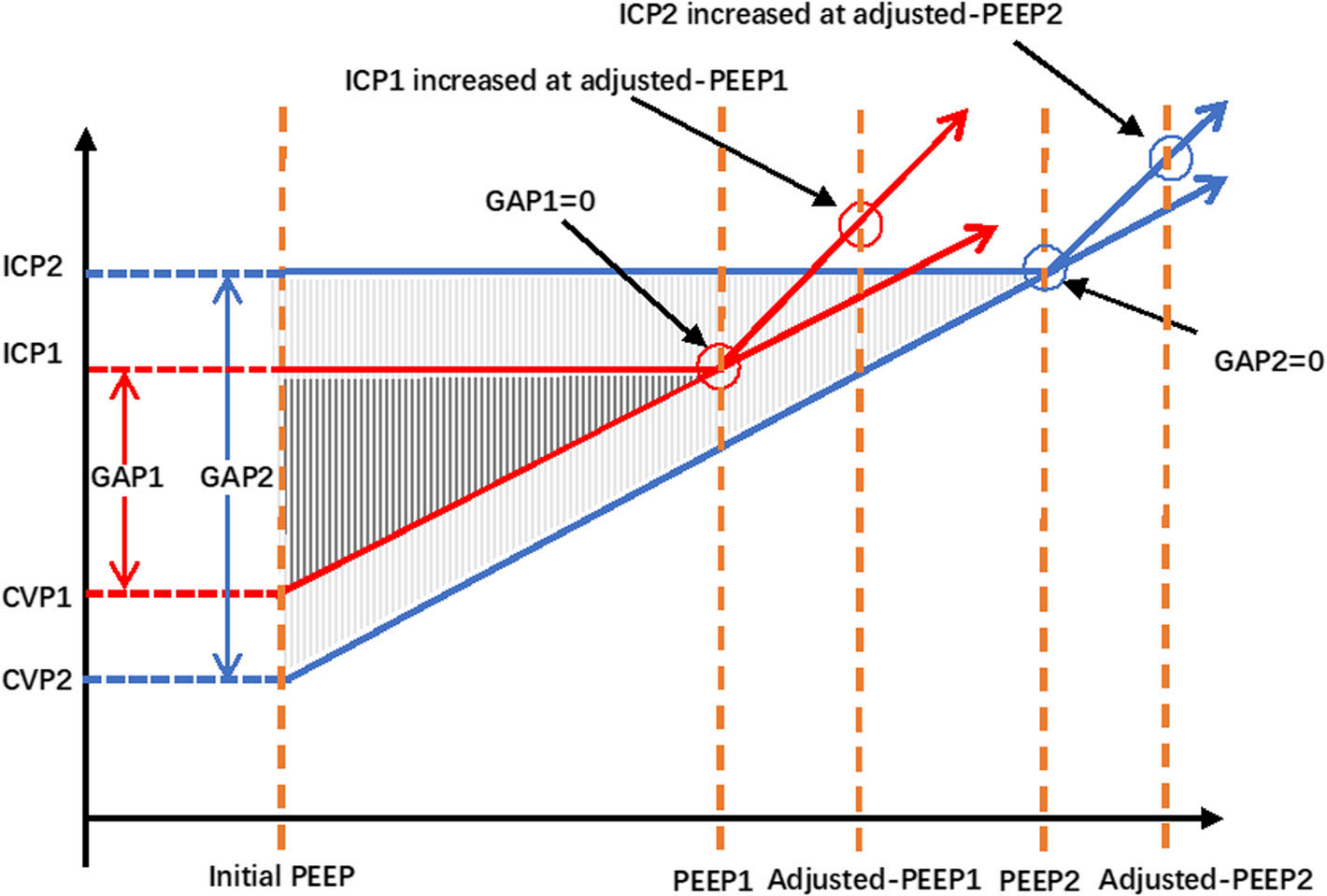
Schematic diagram of the research hypothesis. CVP1 and CVP2 increased from baseline values when PEEP was elevated from initial PEEP; however, ICP1 and ICP2 remained unchanged in the beginning. Thus, GAP1 and GAP2 were narrowed gradually until they disappeared when PEEP reached PEEP1 and PEEP2 (dark grey and light grey shades). PEEP1 and PEEP2 were critical pressure values (GAP1 = 0 and GAP2 = 0) for patient A and patient B, respectively; thereafter, CVP1 at adjusted-PEEP1 would exceed baseline ICP, which would contribute to an elevation of ICP1, and likewise for CVP2 at adjusted-PEEP2. CVP and ICP at initial PEEP were termed baseline ICP and baseline CVP, respectively. ICP1 and CVP1 represented intracranial pressure and central venous pressure at different levels of PEEP for patient A. ICP2 and CVP2 referred to intracranial pressure and central venous pressure for patient B. GAP1 and GAP2 were calculated by ICP1-CVP1 and ICP2-CVP2. Abbreviations: CVP, central venous pressure; PEEP, positive end-expiratory pressure; ICP: intracranial pressure; P_IC_Gap: Gap between baseline ICP and baseline CVP

To date, no indicators for predicting the influence of PEEP on ICP have been reported. Theoretically, increasing intrathoracic pressure by PEEP may hinder cerebral venous return and increase ICP during MV in patients with sTBI, and the relationship between CVP and PEEP has been clarified [16, 17]. Therefore, we hypothesized that: (1) CVP could act as a mediator between PEEP and ICP; (2) the effect of PEEP on ICP depends on baseline ICP and baseline CVP according to the Starling resistor model (Fig. 1). Herein, we developed a new indicator, P_IC_Gap, which represents the difference between baseline ICP and baseline CVP (at initial PEEP). The main aim of this study was to explore the predictive efficiency of P_IC_Gap in ICP responsiveness to PEEP adjustment.

## Methods

### Study design and setting

This prospective study was conducted between May 2016 and May 2019 in the intensive care unit (ICU) of Zhoupu Hospital affiliated to Shanghai University of Medicine & Health Sciences. It has been registered at ClinicalTrials.gov (NCT03296293), and approval of the study protocol was granted by the clinical research ethics committee of Zhoupu Hospital (no. ZPYYLL-2016-12). Written informed consent was obtained from the legal representatives of all patients upon admission because of the coma state of the enrolled study patients.

### Study participants

All patients with sTBI (Glasgow Coma Scale [GCS] ≤8) diagnosed via head CT scan, ICP monitoring transducers or catheter, hypoxemia (pulse oxygen saturation [SpO_2_] < 90%) and MV (Dräger Infinity C500, Dräger, Germany) were initially included. The patients whose hypoxemia could not be corrected even by increasing the fraction of inspired oxygen (FiO_2_) by more than 60% in combination with suction and intensive airway management were eventually enrolled. Exclusion criteria included brain death, age below 18 or over 80 years, pregnancy in women, hemodynamic instability (heart rate [HR] > 120 bpm or CPP [calculated as MAP-ICP] < 60 mmHg), pneumothorax, pulmonary bulla, acute myocardial infarction (elevated cardiac troponin T more than 3 times the normal upper limit accompanied by the ST-T change) and neurosurgical intervention.

### Treatment and data measurements

The treatment process followed the Guidelines for the Management of sTBI [18]. All patients were placed in the supine position at 30 degrees head of bed elevation and deeply sedated (0.05 mg/kg loading dose, followed by continuous intravenous infusion of midazolam at 0.05–0.3 mg/kg/h and sufentanil at 0.2 μg/kg/h) to maintain a Richmond Agitation-Sedation Scale (RASS) score of − 5 and, thus, to remove the effects of cough and other neuronal and confounding factors on ICP. The ventilator settings remained consistent for all enrolled patients. The tidal volume was adjusted and maintained at 8 mL/kg of the predicted body weight, and the plateau pressure was maintained below 30 cmH_2_O. Support pressure was maintained at 12–14 cmH_2_O; initial PEEP was set at 3 cmH_2_O, and FiO_2_ was set at 35–50% to maintain a SpO_2_ 90%. ICP was continuously monitored (Codman ICP ExpressTM, Johnson, USA) through an intraparenchimal transducer or a ventricular catheter (Codman ICP Transducer, Johnson, USA) that was associated with a closed external ventricular drain in each measurement when applicable. Both central venous and arterial catheters were inserted to measure intra-arterial mean arterial pressure (MAP) and CVP. CPP was maintained at more than 60–65 mmHg. The static compliance of the respiratory system (Cst) recorded from the ventilator was indexed to the predicted body weight of the patient. During the study, end-tidal carbon dioxide pressure (PetCO_2_) (monitoring by Drager Mainstream CO_2_ device, SN: ASHM-0552, Dräger, Germany) was maintained at 30–35 mmHg by adjusting tidal volume and the respiratory rate, in order to avoid any effect of CO_2_ on ICP [19].

The stepwise increase of PEEP was set according to a method by Lim et al. [20] when hypoxemia persisted. Briefly, 100%-FiO_2_ was set, and PEEP was increased stepwise (from 3 cmH_2_O to 10 cmH_2_O, and to 15 cmH_2_O) every 2 min, which was a recruitment maneuver known as “extended sigh”. ICP, CVP, Cst, PetCO_2_, and CPP at both levels of PEEP (3 cmH_2_O and 15 cmH_2_O) were measured, respectively. After PEEP at 15 cmH_2_O was maintained for 2 min, baseline ventilator setting was resumed.

Based on our research hypothesis and the specific relationship between CVP and PEEP [16, 17], P_IC_Gap and other measurements (Cst, CPP, ICP, and PetCO_2_) were compared between the responder and non-responder groups, and prediction of ICP responsiveness to PEEP was tested by assessing the area under the receiver operating characteristic (ROC) curve (AUC).

In this study, treatment was provided in case of: (1) CPP < 60 mmHg (norepinephrine 0.3–1.0 μg/kg/min); (2) ICP > 25 mmHg (PEEP was restored to 0); (3) increase in pressure plateau > 35 cmH_2_O (tidal volume was decreased and the respiratory rate was increased to maintain PetCO_2_ at 30–35 mmHg); (4) SpO_2_ declined progressively (PEEP was restored to 0); and (5) suspicion of pneumothorax (PEEP was restored to 0, and chest radiography was undertaken). An equilibration period (≥ 90s) was entailed to ensure a normalized baseline PetCO_2_ through tidal volume and respiratory rate modulation, as described by Flexman and colleagues [9].

### Study outcomes

The main outcome was ICP responsiveness to PEEP adjustment from 3 cmH_2_O to 15 cmH_2_O. Because there is no specified definition for ICP responsiveness, we stipulated that response and non-response referred to increments greater than and less than 20% of baseline ICP, respectively. The patients were then classified into the responder and non-responder groups accordingly.

### Statistical analysis

Categorical variables were presented as number and percentage, and analyzed by Fisher’s exact test. Continuous covariates, including hemodynamic variables ICP, CVP, CPP, Cst, PetCO_2_ and CPP, were expressed as mean ± standard error. One-way analysis of variance (ANOVA) followed by Bonferroni post hoc test was used for multiple comparisons. The predictive roles of P_IC_Gap other related parameters recorded for ICP responsiveness to PEEP were tested by calculating the AUCs of the ROC curves of ICP over the baseline value at two levels of PEEP (3 and 15 cmH_2_O). *P* <  0.05 was considered statistically significant. All statistical analyses were performed with SPSS 20.0 for windows (IBM Co. NY, USA).

## Results

### Patients baseline characteristics between responder group and non-responder group

From May 2016 to May 2019, a total of 174 patients were included initially. Sixty-two patients were excluded for hemodynamic instability neurosurgical interventions before admission of ICU, and hypoxemia corrected by suction and increase of FiO_2_, and 112 were entered into the final analysis (supplementary materials, Figure Flowchart). Baseline characteristics of the study population in the responder (*n* = 49) and non-responder (*n* = 63) groups are shown in Table 1.

**Table 1:** Patients characteristics at PEEP of 3 cmH_2_O between responder group and non-responder group

	Responder group (*n* = 49)	Non-Responder group (*n* = 63)	*p*
Male, n (%)	31 (41.89)	43 (58.11)	0.688
Age, years, mean (SD)	46.96 (11.88)	49.02 (10.49)	0.334
Causes of brain injury, n (%)			
Cerebral contusion	28 (57.1)	38 (60.3)	0.735
Parenchymal hematoma	14 (28.6)	17 (27.0)	0.852
Subdural hematoma	7 (14.3)	8 (12.7)	0.807
GCS, mean (SD)	5.43 (1.61)	5.16 (1.35)	0.336
Hemodynamics variables			
CVP, mmHg, mean (SD)	8.18 (2.66)	6.54 (2.59)	0.001
MAP, mmHg, mean (SD)	78.00 (5.55)	79.60 (4.57)	0.097
ICP, mmHg, mean (SD)	9.82 (2.97)	13.10 (2.74)	< 0.001
CPP, mmHg, mean (SD)	66.67 (4.58)	66.51 (4.03)	0.840
HR, bpm, mean (SD)	74.97 (13.36)	78.88 (14.47)	0.104
P_IC_Gap, mmHg	1.63 (1.33)	6.55 (2.46)	< 0.001
PetCO_2_, mmHg	33.00 (3.13)	33.05 (3.06)	0.931
CrsI, ml/kg/cmH_2_O	1.30 (0.06)	1.31 (0.06)	0.865

Abbreviations: *GCS* Glasgow coma score; *CVP* Central venous pressure; *MAP* Mean arterial pressure; *HR* Heart rate; *ICP* Intracranial pressure; *CPP* Cerebral perfusion pressure; *PetCO*_*2*_ End-tidal carbon dioxide pressure; *CstI* The static compliance of respiratory system (Cst) indexed to the predicted body weight of the patients

Compared with the non-responder group, the responder group had lower P_IC_Gap (1.63 ± 1.33 versus 6.56 ± 2.46 mmHg; *p* <  0.001), lower baseline ICP (9.82 ± 2.97 versus 13.10 ± 2.74 mmHg, *p* < 0.001), and higher baseline CVP (8.18 ± 2.66 versus 6.54 ± 2.59 mmHg, *p* = 0.001).

### Effects of PEEP adjustment on CVP, CVP increment and ICP in the responder and non-responder groups

**Fig. 2:**
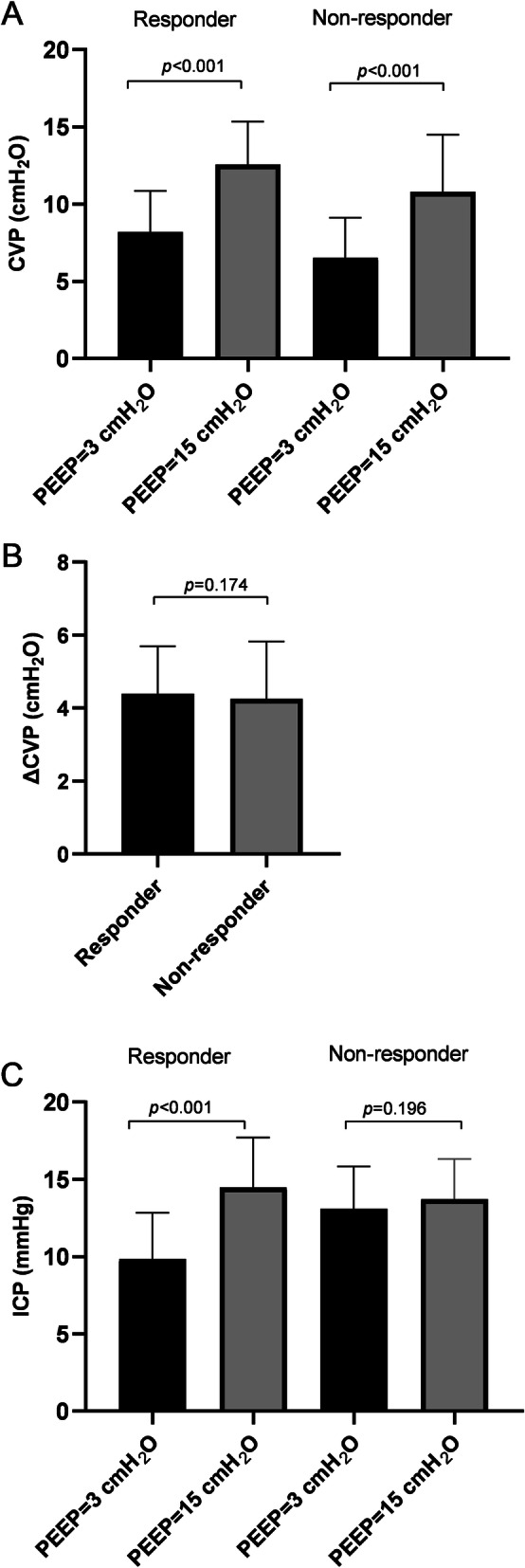
Effects of PEEP adjustment on CVP and ICP in the responder and non-responder groups. Adjustment of PEEP from 3 to 15 cmH_2_O increased CVP significantly in both groups (**a**). There was no significantly difference in CVP increment (ΔCVP) between the responder and non-responder groups (**b**). A significant ICP increase was observed in the responder group with PEEP tuned up from 3 cmH_2_O to 15 mmHg, and no change was found in the non-responder group (**c**). Abbreviations: PEEP, positive end-expiratory pressure; ICP, intracranial pressure; CVP, central venous pressure

Adjustment of PEEP from 3 to 15 cmH_2_O increased CVP levels significantly in both groups (Fig. 2a). There were no significantly differences in CVP increment (ΔCVP) between the responder and non-responder groups (4.39 ± 1.30 versus 4.25 ± 1.58 mmHg; *p* = 0.174) (Fig. 2b). A significant ICP increase was observed in the responder group with PEEP tuned up from 3 cmH_2_O to 15 mmHg (9.85 ± 2.99 versus 14.48 ± 3.22 mmHg; *p* < 0.001), with no change in the non-responder group (13.10 ± 2.74 versus 13.71 ± 2.61 mmHg; *p* = 0.196) (Fig. 2c).

No severe side effects in terms of ICP, CVP, CPP, and MAP were observed when PEEP was increased to 15 cmH_2_O (supplementary materials, Table E1).

### Predictive role of P_IC_Gap, baseline ICP, and baseline CVP on the responsiveness of ICP to PEEP adjustment

**Fig. 3:**
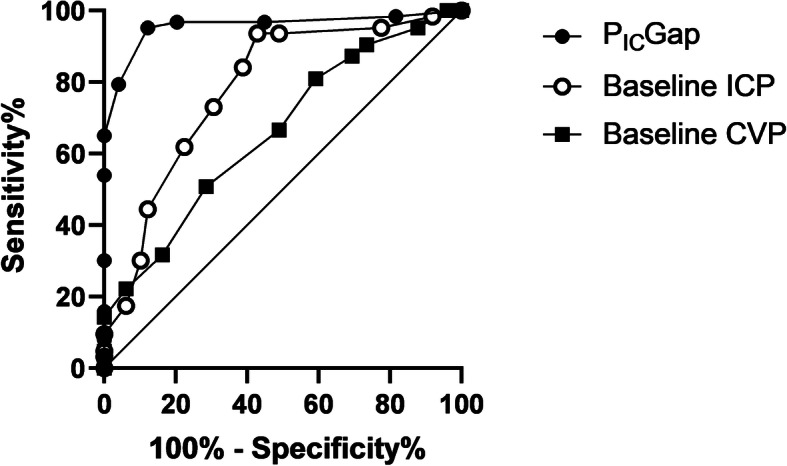
Predictive values of P_IC_Gap, baseline ICP, and baseline CVP for ICP responsiveness to PEEP. Areas under the ROC curves (AUCs) were assessed for various parameters potentially predicting ICP responsiveness to PEEP adjustment. P_IC_Gap had the strongest ability to predict ICP responsiveness to PEEP increase (AUC = 0.957, 95%CI 0.918–0.996; *p* < 0.001) among the three parameters. Meanwhile, baseline ICP had overtly weaker predictive ability than P_IC_Gap (AUC = 0.782 for baseline ICP, 95%CI 0.693–0.781; *p* < 0.001), and baseline CVP had the weakest ability to predict the responsiveness (AUC = 0.660 for CVP, 95%CI 0.560–0.760; *p* = 0.004). Abbreviations: P_IC_Gap, gap between baseline ICP and baseline CVP; ICP, intracranial pressure; AUC, area under receiver operating characteristic curve; ROC, receiver operating characteristic

As shown in Table 1, P_IC_Gap, baseline ICP, and baseline CVP were significantly different between the responder and non-responder groups, and no significant differences were found in the other variables tested. The predictive abilities of P_IC_Gap, baseline ICP, and baseline CVP were assessed through ROC analysis. As shown in Fig. 3, P_IC_Gap had the strongest predictive ability for ICP responsiveness to PEEP increase (AUC = 0.957, 95%CI 0.918–0.996; *p* < 0.001) among the three parameters. At a cut off value of 2.5 mmHg, P_IC_Gap had favorable sensitivity (95.24, 95%CI 86.91–98.70%) and specificity (87.6, 95%CI 75.76–94.27%) in predicting ICP responsiveness to PEEP. However, baseline ICP had an overtly weaker predictive ability compared with P_IC_Gap (AUC = 0.782, 95%CI 0.693–0.781; *p* < 0.001), and baseline CVP had the weakest ability for predicting ICP responsiveness among the three parameters (AUC = 0.660, 95%CI 0.560–0.760; *p* = 0.004).

## Discussion

In the present study, in agreement with our hypothesis that CVP may be an intermediary that delivers pressure from PEEP to ICP, we found that ICP was increased after PEEP only with baseline ICP close to CVP, i.e. P_IC_Gap was narrower in the responder group compared with the non-responder group (1.63 ± 1.33 versus 6.55 ± 2.46 mmHg). This indicates that the same increment of CVP (4.39 ± 1.30 versus 4.25 ± 1.58 mmHg) could obliterate P_IC_Gap in the responder group, but not in non-responders (Fig. 1). We also evaluated whether P_IC_Gap, baseline ICP, and baseline CVP could predict ICP responsiveness to PEEP in patients with sTBI. The results suggested that P_IC_Gap was the strongest predictive indicator among the three parameters. P_IC_Gap less than 2.5 mmHg could predict ICP responsiveness to PEEP tuned up to 15 cmH_2_O. To the best of our best knowledge, this is the first study demonstrating that PEEP-induced changes of ICP depend on P_IC_Gap rather than PEEP itself.

Although cerebral hemodynamics is not governed entirely by extradural venous pressure since normal ICP (8–13 mmHg) is higher than the venous pressure outside the dura (0–5 mmHg), the changes of extradural venous pressure transfer to the brain circulation might rely on a certain situation [21]. The degree of subdural venous collapse was related to the difference between ICP and extradural venous pressure, and this passive collapse acts as a variable venous outflow resistance. An alteration of extradural venous pressure causes up- or downregulation of venous outflow resistance through the self-regulation of the degree of passive collapse according to the Starling resistor model [22].

CVP could act as a surrogate marker of extradural venous pressure, because the pressure falling on the jugular vein is negligible in the supine position. A preliminary experiment also showed that CVP values were the same as those of jugular bulb pressure. According to the Starling resistor model [22], once the value of CVP after PEEP exceeds baseline ICP, venous outflow resistance would be downregulated to the lower limit. In such a situation, the brain circulation would be impeded, with ICP rising accordingly.

The relationship between PEEP and CVP has been validated by previous studies. Stepwise PEEP elevation induces an increase of CVP [17]. An increase of 12 cmH_2_O in PEEP caused a more than 4 mmHg rise of CVP in the current study, which was consistent with previous findings [17]. Thus, it is reasonable to infer that PEEP directly increases CVP, and whether CVP after PEEP could increase ICP depends on the extent of P_IC_Gap narrowing by CVP.

The lower the P_IC_Gap, the easier it is for CVP after PEEP to exceed baseline ICP; then, the Starling resistor would lose effectiveness as a result of the elimination of venous outflow resistance. As indicated in Table 1, patients with responsiveness to PEEP adjustment had relatively lower P_IC_Gap compared with the non-responder group. Thus, based on the hypothesis that CVP is an intermediary which connects PEEP to ICP, we found that P_IC_GAP, a new indicator, could provide a rational explanation regarding the underlying mechanism, which also accounts for the individual heterogeneity proposed by Yang and colleagues [15].

Brain compliance is unfavorable in patients with sTBI because of cerebral edema associated with injury. In this case, cerebral venous return impeded by increased CVP after PEEP would contribute to increasing ICP after P_IC_Gap is narrowed to zero. A study by Robba and colleagues investigated the effects of pneumoperitoneum and the Trendelenburg position on ICP in non-brain injured patients (lower ICP), and demonstrated that both increase ICP [23]. There was no significant change in arterial blood pressure and CPP in the study. Although CVP was not monitored, increased ICP might be due to an obstruction of cerebral venous return theoretically [24].

Several studies used baseline ICP to predict the responsiveness of ICP to PEEP, and found that patients with lower baseline ICP have a positive response to various PEEP levels [25, 26]. These results were consistent with our findings. Individuals with elevated mean baseline ICP experienced no significant changes of ICP during PEEP alteration. However, these studies have not clarified that a certain ICP value could predict ICP responsiveness to PEEP. Our results also showed that baseline ICP was not a more efficient predictive indicator compared with P_IC_Gap.

It should be mentioned that ICP responsiveness to PEEP may be influenced by compliance of the respiratory system [27, 28]. Assessment of patients with low-compliance lungs showed that cerebral hemodynamics and ICP are not influenced by the application of PEEP, because less compliance may not transmit the increased pressure to the entire intrathoracic space effectively. In the current study, all the enrolled patients had normal compliance (Table 1). In addition, recent studies have challenged the effects of PEEP on ICP, arguing that PEEP may be more related to eventual changes in hemodynamics or lung compliance than affecting CVP [29–31]. For example, it was proposed that ICP markedly increases after PEEP application, but only in case PEEP induces alveolar hyperinflation with subsequent PaCO_2_ increase [29].

It is known that sTBI patients tend to progress rapidly early after injury; therefore, they were kept in the ICU for 24 h after injury for close monitoring of ICP in this study. To prevent catheter-related infections, the ICP monitoring probe was implanted for no more than 7 days. In case of hypoxemia, increased PEEP was provided for lung recruitment and increased oxygen saturation. The purpose of a PEEP of 15 cmH_2_O during lung recruitment was to avoid the harmful effects of high PEEP (> 20 cmH_2_O), such as worsened hemodynamics and significantly increased ICP. Consequently, no serious complication was recorded in this study (Table E1). In addition, maintaining PEEP at 15 cmH_2_O for 2 min helped achieve pulmonary bloating [20] and normalize PetCO_2_ to avoid the impact of CO_2_ retention on ICP [9]. PEEP in this study did not adversely affect hemodynamics in both patient groups (Table E1), and there was no significant difference in prognosis between the two groups (Table E2).

The limitations of this study should be mentioned. First, the sample size was relatively small for a clinical study. In addition, the effect of PEEP on CBF was not evaluated. However, we maintained PetCO_2_ at the normal level, and ICP elevation was in a permissible range, indicating likely stable CBF. Furthermore, although P_IC_Gap is a dynamic marker, it did not change dramatically in the early stage of TBI in certain patients after neurosurgery, ensuring the predictive value individually.

## Conclusions

The impact of PEEP on ICP depends on the gap between baseline ICP and CVP. P_IC_Gap, a new indicator, could be a potential predictor of ICP responsiveness to PEEP adjustment in patients with sTBI.

## Supplementary information

**Additional file 1: Table S1.** Hemodynamics and respiratory variables at PEEP of 15 cmH_2_O between two groups.

**Additional file 2: Table S2.** Comparison of prognosis between two groups.

## Data Availability

The datasets used and/or analyzed in the present study are available from the corresponding author on reasonable request.

## Abbreviations

MV: Mechanical ventilation
PEEP: Positive end-expiratory pressure
sTBI: Severe traumatic brain injury
ICP: Intracranial pressure
CPP: Cerebral perfusion pressure
CBF: Cerebral blood flow
CVP: Central venous pressure
P_IC_Gap: Gap between baseline ICP and baseline CVP
ICU: Intensive care unit
GCS: Glasgow coma scale
SpO_2_: Pulse oxygen saturation
FiO_2_: Fraction of inspired oxygen
HR: Heart rate
MAP: Mean arterial pressure
RASS: Richmond agitation-sedation scale
Cst: Compliance of the respiratory system
PetCO_2_: End-tidal carbon dioxide pressure
ROC: Receiver operating characteristic
AUC: Area under the receiver operating characteristic curve
