# Supplementary material for: Intracranial-to-central venous pressure gap predicts the responsiveness of intracranial pressure to PEEP in patients with traumatic brain injury: a prospective cohort study
Source: BMC Neurol. 2020 Jun 8;20:234. doi: 10.1186/s12883-020-01764-7 (PMC7276961; doi:10.1186/s12883-020-01764-7)
Supplement: Supplementary file 1 — Additional file 1: Table S1. Hemodynamics and respiratory variables at PEEP of 15 cmH2O between two groups. [file 12883_2020_1764_MOESM1_ESM.docx]

Table E1. Hemodynamics and respiratory variables at PEEP of 15 cmH_2_O between two groups.

|  | Responder group (n = 49) | Non-Responder group (n = 63) | *p* |
| --- | --- | --- | --- |
| Hemodynamics variables |  |  |  |
| CVP, mmHg, mean (SD) | 13.35 (3.01) | 10.76 (3.70) | < 0.001 |
| MAP, mmHg, mean (SD) | 80.18 (5.28) | 78.29 (6.43) | 0.097 |
| HR, bpm, mean (SD) | 79.34 (15.61) | 82.29 (16.54) | 0.084 |
| ICP, mmHg, mean (SD) | 14.45 (3.20) | 13.71 (2.61) | 0.183 |
| CPP, mmHg, mean (SD) | 65.73 (4.58) | 64.57 (5.82) | 0.253 |
| PetCO_2_, mmHg | 33.73 (3.11) | 33.43 (3.07) | 0.604 |
| CrsI, ml/kg/cmH_2_O | 1.30 (0.05) | 1.29 (0.05) | 0.243 |

Abbreviations: CVP, central venous pressure; MAP, mean arterial pressure; HR, heart rate; ICP, intracranial pressure; CPP, cerebral perfusion pressure; PetCO_2,_ end-tidal carbon dioxide pressure; CstI, the static compliance of respiratory system (Cst) indexed to the predicted body weight of the patients.
