# Supplementary material for: Intracranial-to-central venous pressure gap predicts the responsiveness of intracranial pressure to PEEP in patients with traumatic brain injury: a prospective cohort study
Source: BMC Neurol. 2020 Jun 8;20:234. doi: 10.1186/s12883-020-01764-7 (PMC7276961; doi:10.1186/s12883-020-01764-7)
Supplement: Supplementary file 2 — Additional file 2: Table S2. Comparison of prognosis between two groups. [file 12883_2020_1764_MOESM2_ESM.docx]

Table E2 Comparison of prognosis between two groups

|  | Responder group (n = 49) | Non-Responder group (n = 63) | *p* |
| --- | --- | --- | --- |
| ICU stay, day, mean (SD) | 12.41(2.16) | 13.69(2.24) | 0.104 |
| MV duration, day, mean (SD) | 6.35 (3.20) | 7.76 (3.91) | 0.079 |
| Neurosurgical interventions, n, (%) | 11 (22.44) | 16(25.40) | 0.096 |
| Mortality of 28 days, n, (%) | 5 (10.20) | 7(11.11) | 0.871 |

Abbreviations: ICU, intensive care unit; MV, mechanical ventilation
